# Primary orbital ganglioneuroblastoma: A case report

**DOI:** 10.1515/med-2021-0230

**Published:** 2021-07-15

**Authors:** Ruixin Ma, Yujiao Wang, Weimin He

**Affiliations:** Department of Ophthalmology, Ophthalmic Laboratory, West China Hospital, Sichuan University, Chengdu 610041, Sichuan, China

**Keywords:** primary ganglioneuroblastoma, orbit, adrenal gland

## Abstract

**Background:**

Ganglioneuroblastoma (GNB) is a neoplasm that arises from the primitive cells of the sympathetic nervous system during childhood. The current case is very unique because of the initial primary tumor manifestation in the orbit and an adrenal tumor being observed later during follow-up.

**Case presentation:**

A 2-year-old girl presented to the Ophthalmology Department of our hospital complaining of swelling of the left upper eyelid for approximately one month. Orbital computed tomography (CT) revealed a left orbital mass with bone destruction. Thoracic and abdominal CT indicated no abnormalities. The mass was surgically resected, and histopathological analysis confirmed it as GNB. During follow-up, abdominal CT detected an adrenal tumor with internal calcification, a calcified nodule on the left side of the abdominal aorta, and mesenteric lymph nodes. Accordingly, primary orbital GNB and metastatic adrenal GNB were the possible considerations. We removed the adrenal tumor, and the patient underwent chemotherapy. However, the patient died 18 months after the ophthalmic surgery.

**Conclusion:**

Primary orbital GNB in children is easily misdiagnosed because of its rare occurrence and atypical clinical findings. Imaging methods combined with histopathological examination contribute to the detection and diagnosis of primary and metastatic GNBs. Thus, timely surgery combined with adjuvant chemotherapy and long-term follow-up is essential for controlling the metastasis of GNB and improving the survival rate of patients.

## 1 Introduction

Ganglioneuroblastoma (GNB) is a neuroblastic tumor originating from the primordial neural crest cells in the sympathetic nervous system and is frequently observed in the pediatric population [1]. GNB, first reported by Wahl and Craig, is a histological subgroup of neuroblastomas and has an intermediate risk of malignancy compared with ganglioneuromas and neuroblastomas [2,3]. The clinical behavior of GNB is unpredictable as the tumor may regress spontaneously, mature into ganglioneuroma, or metastasize rapidly as observed in neuroblastoma. The common sites of GNB are the adrenal gland (35%) and retroperitoneum (30%), whereas metastases usually occur in the bone marrow (70.5%) and the bone (30.9%) [4]. To the best of our knowledge, there are four case reports on the orbital GNB [5,6,7,8]. However, primary orbital GNB has never been reported previously. Herein, we described a rare case wherein a primary GNB was detected in the orbit and a metastatic adrenal tumor was found later during follow-up.

## 2 Case presentation

A 2-year-old girl was admitted to the Ophthalmology Department of our hospital with a swelling of the left upper eyelid for approximately one month. No redness in the eye or other abnormal symptoms were observed. There were no pregnancy or childbirth-related abnormalities reported by the patient’s mother. Also, no familial anamnesis of cancer existed.

**Figure 1:**
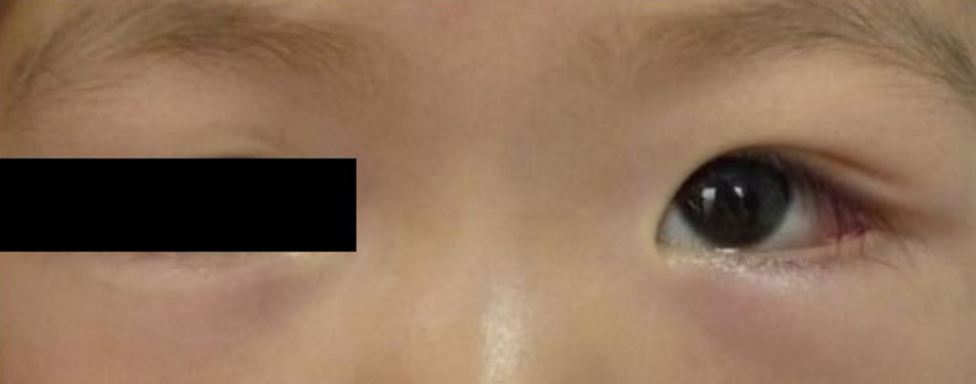
The ocular appearance shows swelling of the lateral superior part and the lower eyelid of the left eye.

**Figure 2:**
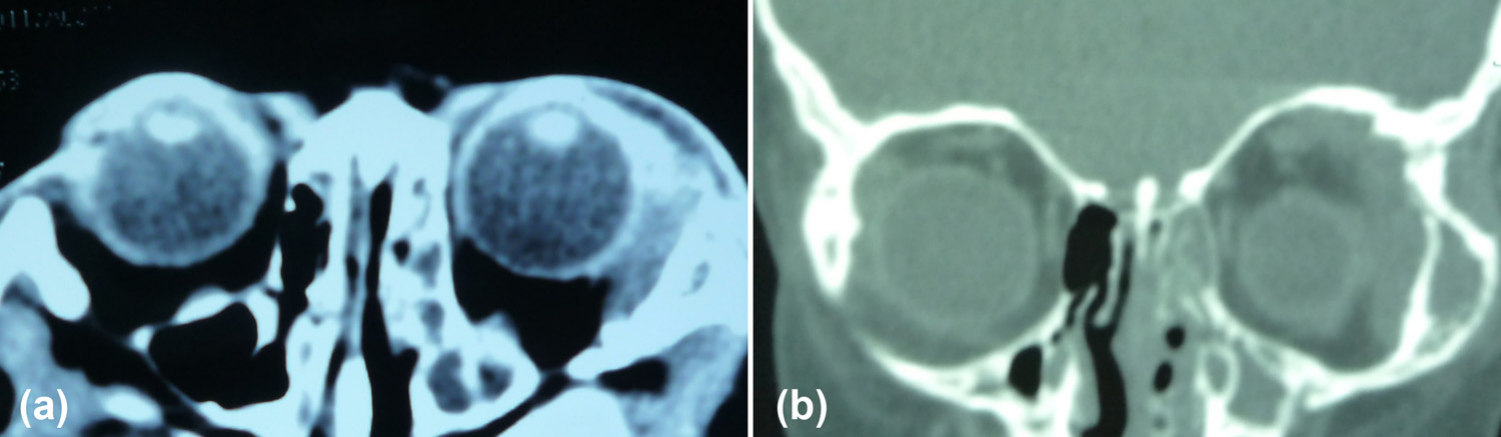
The radiological findings of the 2-year-old girl with an orbital mass. (a) Orbital axial CT showing the soft tissue mass on the lateral orbital rim of the left eye; (b) orbital coronal CT in the bone window showing the soft tissue mass on the lateral superior orbital rim of the left eye, with bone destruction in the lateral wall of the left orbit.

During the initial clinical assessment, the patient had normal growth and good nutritional status and had no abdominal symptoms such as abdominal pain and abdominal swelling. Thoracic and abdominal CT revealed no detectable abdominal masses. On ophthalmic examination, a mass on the lateral superior orbital rim of the left eye, with congestion and edema of the superotemporal conjunctiva and swelling of the lower eyelid, was detected (Figure 1). Orbital CT showed a soft tissue mass on the lateral superior orbital rim of the left eye with bone destruction in the lateral wall of the left orbit (Figure 2).

**Figure 3:**
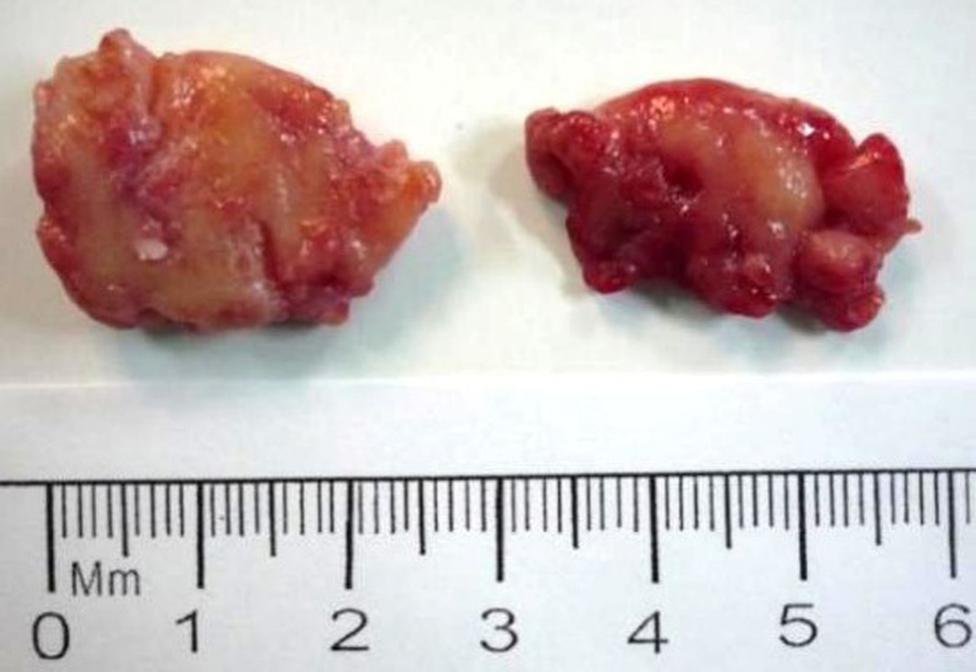
The gross picture shows two masses measuring approximately 2.5 cm × 2 cm × 1.5 cm and 2.5 cm × 1.5 cm × 1 cm in size.

**Figure 4:**
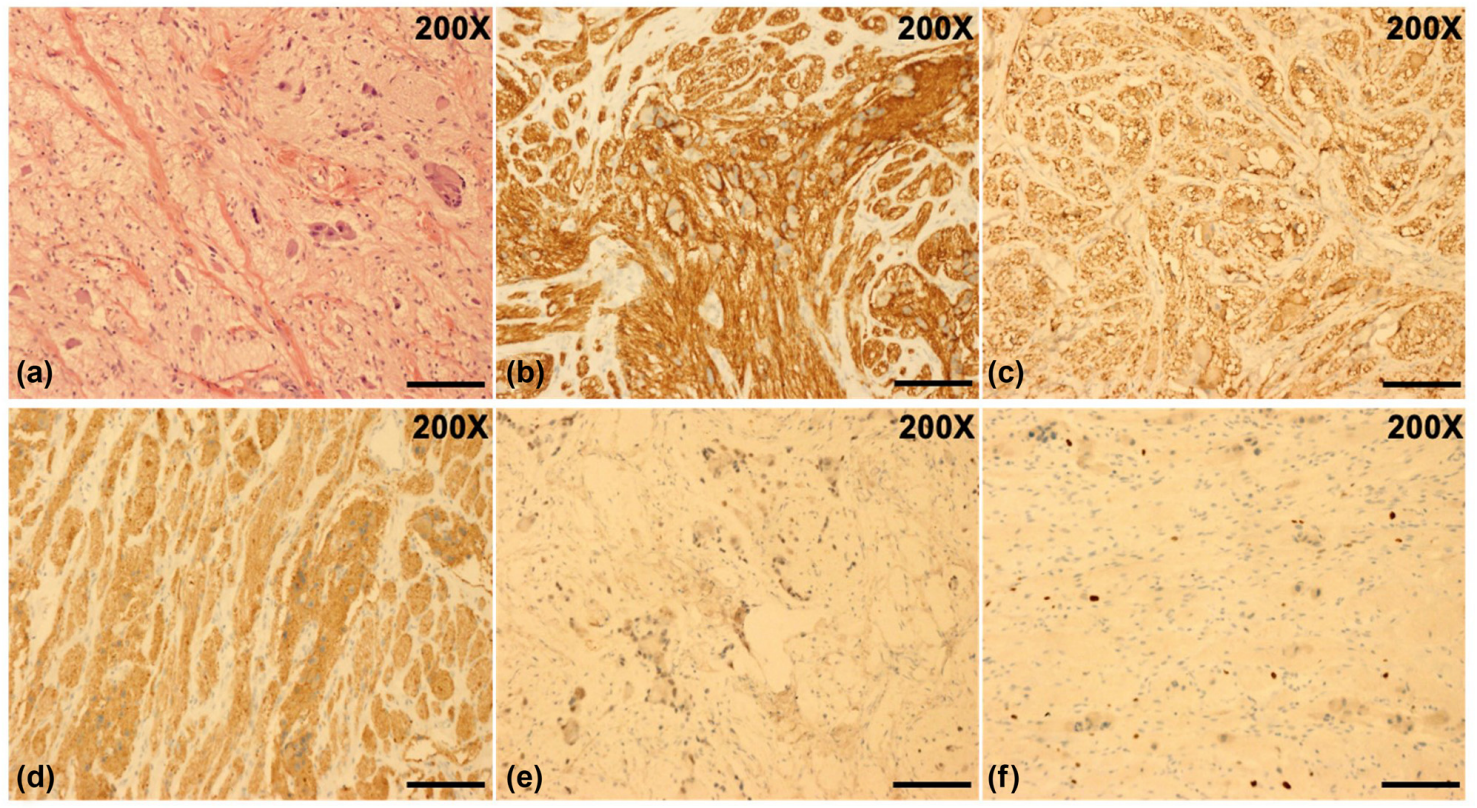
Histopathological examination of the tumor. (a) Hematoxylin-eosin (H&E) staining results of the tumor showed neuroblasts at different degrees of differentiation arranged in nests and mature gangliocytes with fibrous connective tissue septa (H&E, original magnification, 200×); (b) the number of CD56-positive tumor cells was 175.7 ± 6.5 per 3 high power field (HPF) (CD56, original magnification, 200×); (c) immunohistochemical staining results were positive for NSE and the number of these tumor cells was 163.3 ± 5.6 per 3 HPF (NSE, original magnification, 200×); (d) the number of Syn-positive tumor cells was 172.1 ± 25.2 per 3 HPF (Syn, original magnification, 200×); (e) the number of NeuN-positive cells was 122.7 ± 11.6 per 3 HPF (NeuN, original magnification, 200×); (f) the proportion of Ki-67-positive cells was 2–5% per HPF (Ki-67, original magnification, 200×). Scale bar = 200 μm. ImageJ 1.50b with cell counter plugin (https://imagej.nih.gov/ij/) was used for cell counting following the online guide.

**Figure 5:**
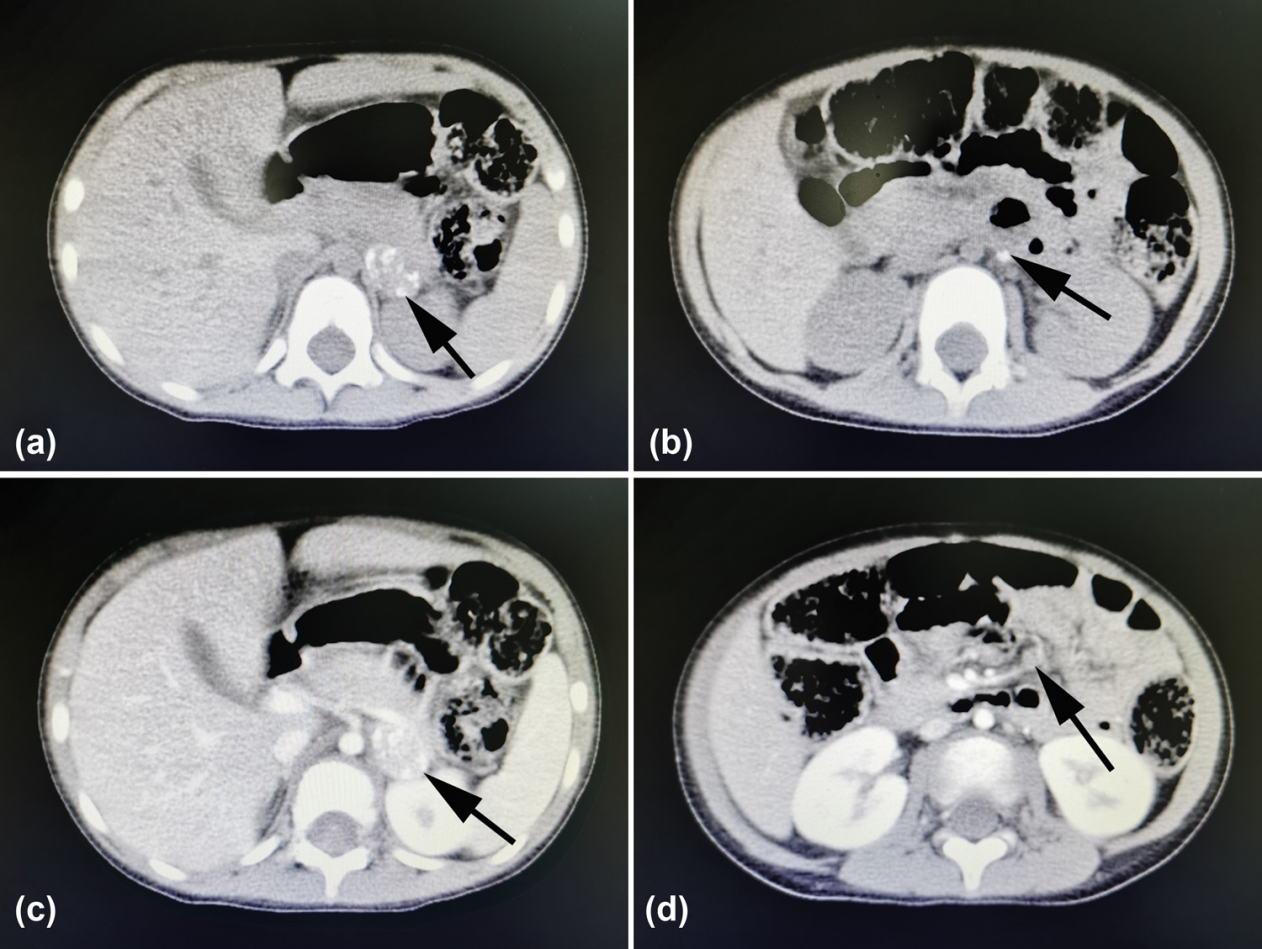
Abdominal CT findings. (a) A plain scan showing a left adrenal tumor of approximately 1.5 cm × 2.2 cm × 2.7 cm size (black arrow); (b) a plain scan showing a calcified nodule on the left side of the abdominal aorta (black arrow); (c) an enhanced scan revealing left adrenal tumor with increased calcification inside (black arrow); (d) an enhanced scan showing the mesenteric lymph nodes (black arrow).

To determine the composition of the left orbital mass, the patient underwent a resection surgery under general anesthesia. During the surgery, we found that the mass was growing close to the lateral and superior wall of the orbit and showed a cord-like shape, dark red color, and an irregular surface. It adhered extensively to the surrounding tissues, with adjacent bones widely destroyed. The mass was removed as much as possible. On a general observation, the two tumor masses measured approximately 2.5 cm × 2 cm × 1.5 cm and 2.5 cm × 1.5 cm × 1 cm (Figure 3). Postoperative histopathological analysis revealed neuroblasts in different differentiation stages arranged in nests and mature gangliocytes, with fibrous connective tissue septa. Immunohistochemical staining results were positive for CD56, neuron-specific enolase (NSE), and synaptophysin (Syn), partly positive for neuron-specific nuclear protein (NeuN) and Ki-67 (2–5%), and negative for leukocyte common antigen (LCA), confirming a diagnosis of GNB (Figure 4). After surgery, although the doctor suggested that the patient undergo further chemotherapy or radiotherapy, the patient’s parents refused. During one month follow-up period after surgery, abdominal CT indicated a 1.5 cm × 2.2 cm × 2.7 cm mass with calcification in the left adrenal gland, a calcified nodule on the left side of the abdominal aorta, and several mesenteric lymph nodes (Figure 5). Subsequently, the previously observed ocular symptoms recurred in the operated eye two months after surgery. The patient was scheduled for regular chemotherapy including arsenicals combined with cyclophosphamide, adriamycin, and vincristine. However, at five months after chemotherapy, the patient was not doing well, and frontal bone and rib destruction was detected on a bone scintigraphy. The adrenal tumor was removed by surgery and was histologically confirmed to be GNB. Postoperative adjuvant chemotherapy was initiated. Unfortunately, at eight months after the adrenal surgery, the patient died of bone metastasis. Informed consent was obtained from the patient’s parents for publishing the study.

## 3 Discussion

Neuroblastic tumors are the most common childhood solid extracranial tumors, and GNB constitutes 20% of these tumors [9]. Clinical symptoms of patients with orbital GNB include Horner’s syndrome and periorbital bony lesions that destroy the palpebral vessels draining the periorbital soft tissue [10,11]. Thus, patients presenting with periorbital ecchymosis and edema called “raccoon eyes” account for 30% of all orbital GNB patients [12]. However, “raccoon eyes” is a nonspecific symptom. Skull base fractures, Kaposi’s sarcoma, multiple myeloma, and amyloidosis can also present “raccoon eyes.” Thus, imaging and pathological differential diagnosis are indispensable for confirming GNB. Imaging examinations, such as CT, magnetic resonance imaging (MRI), and ultrasonography, can be used to find neuroblastic tumors. On plain CT, a tumor usually shows an irregular soft tissue mass, unclear edge, no obvious capsule, proneness to cystic change, and necrosis, accompanied by calcification and invasion to the surrounding tissues [13]. However, because of the atypical clinical characteristics and imaging features of CT, histopathological examination plays a significant role in the definitive diagnosis of neuroblastoma subtypes, in differential diagnosis, and in the determination of cellular maturation degree. These tumors vary in their relative proportion of neuroblasts and Schwann cells. Undifferentiated neoplasms are composed almost entirely of immature and undifferentiated neuroblasts. Differentiated cells include neuroblasts differentiating into ganglion cells and mature ganglion cells. Malignant neuroblastoma shows less than 50% differentiated elements, with poor stroma and thin fibrovascular septa. Homer Wright rosette is a typical characteristic of a neuroblastoma, with circular or ovoid columns of tumor cells arranged around a central core of the neuropil. However, it is not always present. Benign ganglioneuroma comprises 100% differentiated cells, with a dominant stroma characterized by extensive growth of Schwann cells. GNB is intermediate, with more than 50% differentiated cells, as was also observed in this case [2,14,15]. GNB can secrete catecholamines, such as the vanillylmandelic acid (VMA) and homovanillic acid (HVA). Furthermore, the VMA-to-HVA ratio is often used as an indicator of tumor maturity [16]. Moreover, NSE, CD56, Syn, and chromogranin A (CgA) are the frequently used neuronal markers and have high specificity; their positive rates are indicative of the severity of neuroblastomas [17,18]. Furthermore, it is the increased amplification and expression of MYCN, a proto-oncogene, and it always predicts poor prognosis [19,20]. In the presented case, the immunohistochemical results for NSE, CD56, and Syn were positive and those for NeuN were partly positive, favoring the diagnosis of GNB.

In 60–70% of cases, metastases are present with primary lesions at the initial diagnosis [9]. Two cases have been previously reported wherein GNB originated from the adrenal gland and metastasized to the orbit, and in both these cases, the primary and metastatic sites were diagnosed simultaneously [7,8]. However, in our case, only the left orbital mass with bone destruction was found on orbital and abdominal CT at the initial visits. Abdominal CT conducted during the postoperative follow-up revealed the adrenal tumor, which was pathologically confirmed as GNB. Thus, the primary orbital GNB and metastatic adrenal GNB were considered.

Surgical resection is the mainstay treatment for GNBs, and chemotherapy is recommended for high-risk patients [21,22]. In this case, the primary orbital tumor was timely resected, but postoperative chemotherapy was delayed, which may have been responsible for tumor recurrence and metastasis.

To the best of our knowledge, this is the first reported case of primary orbital GNB and metastatic adrenal GNB. The four previous case studies associated with orbital GNB have been reported abroad, and we could not find similar domestic studies on literature review. Two of these studies reported cases with both adrenal GNB and orbital GNB. One study reported the case of a 10-month-old girl with metastatic orbital GNB and primary adrenal GNB. She underwent chemotherapy and survived after 7 years of follow-up. Another study reported a 2-year-old girl with primary adrenal GNB and metastatic lesions including the calva, orbit, and bone marrow. She underwent neoadjuvant chemotherapy, surgery, and bone marrow transplantation and survived without recurrence (Table 1).

**Table 1:** Comparison of published cases of orbital ganglioneuroblastoma

Author	Year	Age	Organ involvement	Treatment	Outcome
Kim et al.	2011	2 years	Skull, orbit, adrenal gland, bone marrow	Chemotherapy, surgery, bone marrow transplantation	Survived, no recurrence
Johnson and Toledano	2003	10 months	Adrenal gland, orbit, skull	Chemotherapy	Survived (>7 years)
Dhermy et al.	1985	Not reported			
Salas and Esparza	1963	Not reported			

## 4 Conclusion

Our report highlights the importance of early diagnosis and intervention for primary orbital GNB in children with an orbital bony lesion, especially in cases with a primary orbital tumor with “raccoon eyes.” Imaging methods combined with histopathological examination can contribute to the accurate diagnosis of the primary and metastatic lesions, particularly in the adrenal gland. Furthermore, timely surgery combined with adjuvant chemotherapy and long-term follow-up is essential for reducing the recurrence rate and metastasis of GNB and for improving the survival rate of the patients.
